# Integration of proprioception in upper limb prostheses through non-invasive strategies: a review

**DOI:** 10.1186/s12984-023-01242-4

**Published:** 2023-09-09

**Authors:** Ermanno Donato Papaleo, Marco D’Alonzo, Francesca Fiori, Valeria Piombino, Emma Falato, Fabio Pilato, Alfredo De Liso, Vincenzo Di Lazzaro, Giovanni Di Pino

**Affiliations:** 1 Research Unit of Neurophysiology and Neuroengineering of Human-Technology Interaction (NeXTlab), Università Campus Bio-Medico Di Roma, Via Álvaro Del Portillo 21, 00128 Rome, Italy; 2 Research Unit of Neurology, Department of Medicine and Surgery, Università Campus Bio-Medico Di Roma, Via Alvaro del Portillo, 21, 00128 Rome, Italy; 3 Fondazione Policlinico Universitario Campus Bio-Medico, Via Alvaro del Portillo 200, 00128 Rome, Italy

**Keywords:** Prosthetics, Upper-limb amputation, Proprioception, Non-invasive feedback

## Abstract

Proprioception plays a key role in moving our body dexterously and effortlessly. Nevertheless, the majority of investigations evaluating the benefits of providing supplemental feedback to prosthetics users focus on delivering touch restitution. These studies evaluate the influence of touch sensation in an attempt to improve the controllability of current robotic devices. Contrarily, investigations evaluating the capabilities of proprioceptive supplemental feedback have yet to be comprehensively analyzed to the same extent, marking a major gap in knowledge within the current research climate. The non-invasive strategies employed so far to restitute proprioception are reviewed in this work. In the absence of a clearly superior strategy, approaches employing vibrotactile, electrotactile and skin-stretch stimulation achieved better and more consistent results, considering both kinesthetic and grip force information, compared with other strategies or any incidental feedback. Although emulating the richness of the physiological sensory return through artificial feedback is the primary hurdle, measuring its effects to eventually support the integration of cumbersome and energy intensive hardware into commercial prosthetic devices could represent an even greater challenge. Thus, we analyze the strengths and limitations of previous studies and discuss the possible benefits of coupling objective measures, like neurophysiological parameters, as well as measures of prosthesis embodiment and cognitive load with behavioral measures of performance. Such insights aim to provide additional and collateral outcomes to be considered in the experimental design of future investigations of proprioception restitution that could, in the end, allow researchers to gain a more detailed understanding of possibly similar behavioral results and, thus, support one strategy over another.

## Background

Upper limb amputations affect millions of people worldwide. A recent estimation from Global Burden of Disease studies stated that in 2017 more than 20 million people were living with an upper limb amputation due to traumatic causes, including 11.3 unilateral and 11 bilateral million amputation cases. These represent 19.6% and 19.1%, respectively of the 57.7 million cases considering all levels of amputation reported [1]. While peripheral arterial disease and diabetes account for the majority of lower limb amputations worldwide, the most common causes for upper limbs depend on the geographical region of occurrence [2]. In developing countries, trauma is the primary cause of amputations for people under the age of 50 [3], and in the vast majority of cases (90–92%) it results from industrial accidents [4, 5].

The currently available types of prostheses include cosmetic prostheses, light but not useful for replacing lost motor functions, and functional body-powered and myoelectric prostheses. Conflicting results have been found in terms of the relative performances of the latter group, with no conclusive evidence in favor of one of them [6]. Two out of three upper limb amputees report a high level of dissatisfaction with their current prosthesis [7], both in terms of performance and comfort [8].

Users exploit sounds and vibrations coming from the motors and the torques transmitted by the socket as a source of somatosensory information to control the prosthesis. However, together with slow and noisy mechanics, unsatisfactory wearability, and poor dexterity issues, the lack of purposely delivered sensory feedback represents a critical limitation and reason for the refusal of the device [9, 10]. Not only the lack of feedback affects prosthesis control, but it is also likely to be one of the causes of poor integration of the prosthesis in the body schema of the user, affecting its acceptability and user’s confidence [11, 12, 13].

Sensory feedback includes both exteroceptive senses, providing us information about the environment, and proprioception. The broad spectrum of proprioception includes the senses of position and body movement, together with the sense of the force exerted and objects’ heaviness [14]. Previous works on artificial feedback in prosthetics mostly focused on the restitution of touch, but relatively fewer studies addressed how to provide proprioceptive information. Also, a critical overview of this topic is still missing. Here, after discussing how the neurophysiology of proprioception may be artificially recreated with the available technology, we provide a review of the studies where non-invasive strategies are employed to provide proprioceptive feedback in arm and hand prostheses. We included kinematics and dynamics studies, in which position and motion information alone or supplemented by force information is returned, respectively. Finally, we discuss the main findings and limits of these studies, together with some proposals to be possibly implemented in future studies.

### Natural and artificial: extracting and translating proprioception

**Fig. 1:**
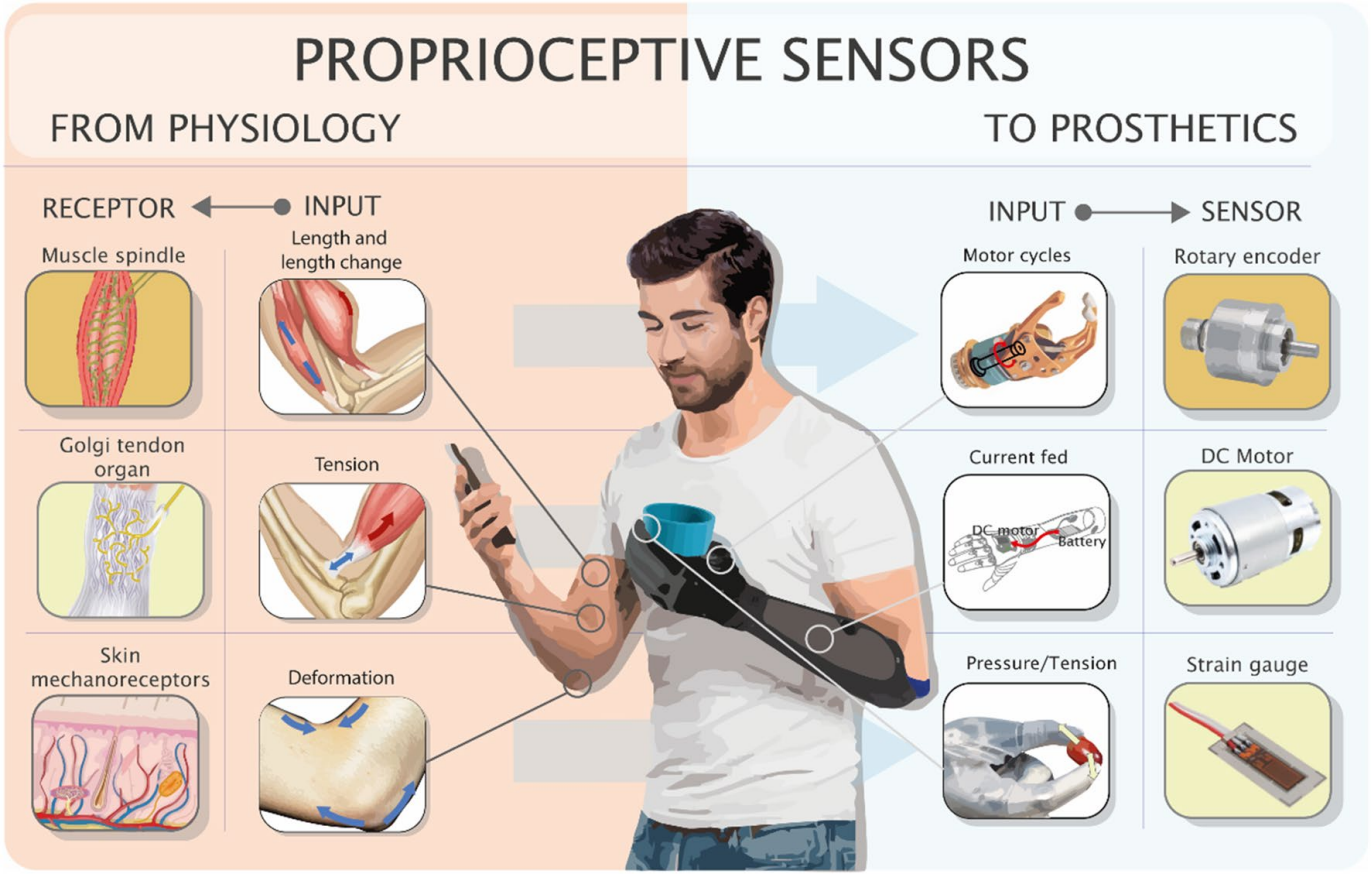
Graphic comparison between physiological and artificial transduction of proprioceptive information. The outer columns show how the function of the receptors in our body (left) could be emulated by some of the common sensors already available on the market (right). In prosthetics, the employed hardware dictates the strategies to be implemented (inner columns) and the input signal may differ from the one coded by the physiological receptor. For instance, the number of cycles of the motors extracted by rotary encoders is used instead of the muscle length, while the current to the motors may replace tendon tension, as an alternative to a tension sensor, which is less common and would need to be purposefully integrated into the device additionally

Developing from the concept of muscular “receptivity”, i.e., the body acts as a stimulus for its own receptors [15], despite the lack of an agreed-upon definition, the most widely accepted has proprioception to include the sense of position and movement, sense of tension or force, sense of effort, and sense of balance [14]. Proprioception is built through the summation of inputs from several peripheral receptors, providing a unique percept. Muscle spindles, however, play a key role in providing body posture and movement, whereas Golgi tendon organs (GTOs) account for the sense of tendon tension and muscle load. Likewise, following the amputation and the loss of these receptors, ideally, the same type of information should be extracted from purposely instrumented prostheses (Fig. 1).

#### Information on position & movement

Muscle spindles are stretch-sensitive mechanical receptors found virtually in all skeletal muscles, except for most facial muscles [16, 17]. Muscle spindles follow length changes of the parent muscle so that the firing rate of their sensory innervation is proportional to the length of the fibers as well as the rate of length change itself. The functional unit of the receptor is formed by a bundle of specialized muscle fibers encapsulated by a connective tissue capsule, called intrafusal fibers, and divided into Bag (Bag1, Bag2), and Nuclear Chain fibers. These receptors, given the length and length change sensitivity, are thought to inform of both position and movement senses [14]. In particular, Group II afferent fibers innervating Bag2 and Nuclear chain fibers are responsible for constant length monitoring, while Group Ia afferents, innervating the fibers of the whole spindle, including Bag1 type, respond also the rate of length change. Such a difference depends also on the type of efferent innervation of the intrafusal fibre, being static gamma-motor neurons for the former group and dynamic gamma-motor neurons for Bag1 fibers [18]. Efferent fusimotor innervation, indeed, elicits the contraction of the polar regions of intrafusal fibers and thereby regulates the tension of the central sensory region [18, 19] (For a detailed description of the mechano-transduction process, refer to [20]). In prosthetics, the sensors employable to extract kinesthetic information depend both on the preferences of the designer, and, most of all, on the actuators (i.e., devices converting energy into motion and force) operating the prosthesis. For instance, incremental magnetic encoders embedded on the motors were employed to provide finger position monitoring, acting like cybernetic hand muscle spindles [21]. In this case, the number of cycles of the motor (the higher the number of cycles, the greater the movement of the end-effector, i.e., the finger) is transduced in place of the muscle length. A biomimetic approach, trying to reproduce muscle spindles artificially, has also been attempted [22, 23] and several mathematical models have been made (i.e., Mileusnic et al., [24]) to simulate the different response dynamics of intrafusal fibers, but to date, no examples of their application in prosthetics are known to the authors.

Cutaneous mechanoreceptors in superficial and deep layers of the skin contribute to kinesthetic senses [25], providing signals to be integrated with the information coming from the spindles of the muscles acting on one joint, as well as information from mechanoreceptors present in the joint itself [26, 27]. Indeed, the movement of a joint is accompanied by a pattern of skin strain and deformation that varies with the speed and amplitude of the movements, which is then translated into neural signals by the mechanoreceptors through their coupling of semirigid connective tissue structures and nervous terminals [28]. Rapidly adapting Meissner and Pacinian corpuscles, are known, for example, to be involved in the detection of finger joints movement [29]. The function of these receptors has been extensively reproduced artificially, both through a biomimetic approach and by using more common electronic sensor technology. However, despite the capability of skin mechanoreceptors to transduce both exteroceptive and proprioceptive movement-related stimuli, artificial counterparts, like strain gauge or piezoelectric sensors, have been mostly employed for providing touch information and for texture recognition, leaving aside the potential application in proprioception substitution [30, 31, 32, 33].

Building on the physiological basis of proprioception is a potential strategy to approach sensory feedback. Spindle afferents can be, indeed, activated through tendon vibration evoking a so-called kinesthetic illusion or tendon-vibration illusion (TVI). Vibration affects spindle afferents, whose firing is entrained to the same rate as the one of the stimuli, creating the sensation of muscle stretch and, thus, the illusion of movement in the direction that would elongate the vibrated muscle [34]. A similar phenomenon also occurs when a mechanical stretch stimulation is applied to the skin close to a joint. These paradigms are currently being investigated as possible strategies to relay prosthesis proprioceptive-like information to the user (e.g., [34], *see below*).

#### Information on force, tension & effort

Golgi tendon organs (GTOs) are mechanoreceptors located in series with the muscle at the transition region between muscle fibers and tendons [36]. Each GTO is innervated by one single Ib large diameter rapidly-conducting afferent fiber, that responds to active tension of the muscles and to changes in contractile force in discrete steps, reflecting the recruitment of additional motor units (motoneuron + innervated muscle fibers) [37]. Sense of effort refers to the sensation experienced when engaging in motor activities, which are directly related to the task being executed and refers to the force muscles need to generate to complete such task. It is thought to be generated centrally, through the transmission of the efference copy [38], an “internal copy” of the motor command. Sense of tension or force (the perception of the amount of external resistance that must be overcome to perform a particular task) comes from the activation of GTO, together with muscle spindles contribution. Finally, evidence suggests that heaviness sensation can be generated either centrally or peripherally, possibly influenced by what the subject focuses their attention on [39].

Either biomimetic or more conventional strategies can be used to sense the force exerted by the prosthesis. In the former case, for example, tension sensors mimicking the behavior of GTOs in the cable transmission have been used to monitor the force applied by a sensorized myoelectric hand [21]. Alternatively, it is possible to measure the current fed to the prosthetic motor and put it in relation to the angular displacement of the end-effector to calculate the contact stiffness [40]. However, in commercial devices, the EMG-driven direct current (DC) motors are not equipped with any intrinsic sensor [41]. Finally, given that the relation between object stiffness and surface deformation is related to the applied pressure, superficial resistive sensors can also be used to indirectly measure the grip force applied by an artificial hand [30].

While a vibrotactile device can generate a kinesthetic sensation by stimulating spindle afferents (i.e., homomodal stimulation, *see: Restitution of Proprioception*), a force and heaviness sensation cannot be elicited in a homomodal way with current technology. This could definitely represent an issue to be addressed in future works.

### Restitution of proprioception

**Fig. 2:**
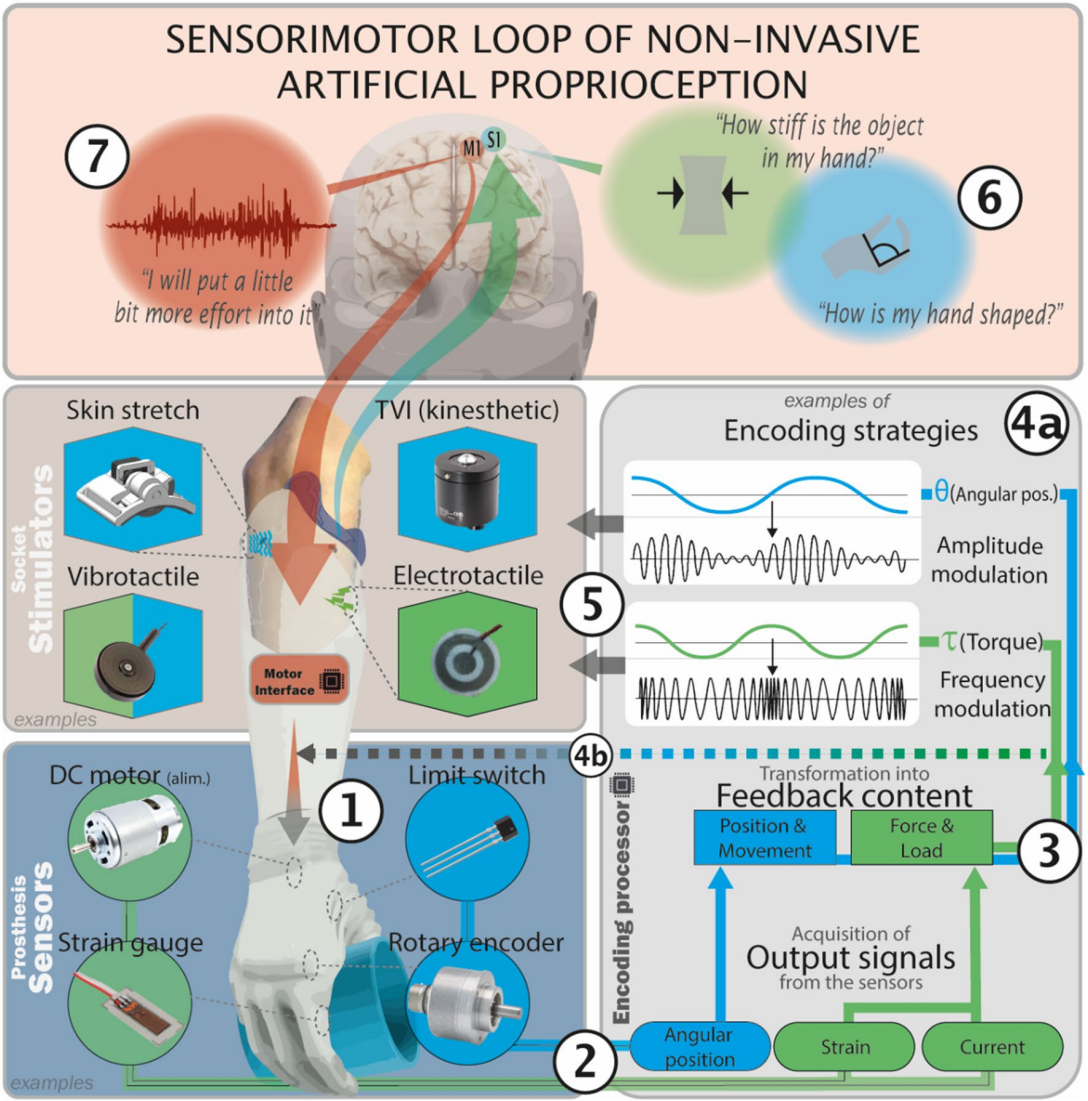
Acquiring and encoding proprioceptive info for sensorimotor integration in prosthetics. 1. The actuator of the prosthesis operates the end effector based on the uptaken biologic signals of the user (e.g., EMG activity through surface electrode in case of myoelectric devices); 2. Sensors embedded in the prosthesis extract the configuration and power developed by the device (e.g., the joint angle is uptaken by the rotary encoders, pressure by superficial sensors and the force exerted is derived from the current adsorbed by the motors), also accordingly with the interaction with the environment (e.g., the cup grasped); 3. Data acquired from the sensors, which refer to proprioceptive-like parameters characterizing the state of the device are translated into feedback content to be delivered to the user; 4a. The feedback content is encoded back into input signals for the stimulators, on the basis of the amount of information to be transmitted, as well as the hardware’s capacities; 4b. The prosthesis, if implemented, can automatically modify (reflex-like behavior) the motor output based on the uptaken data; 5. The stimulators integrated into the device socket deliver the information to the sensory channels available in the stump or elsewhere (e.g., skin-stretch and electrotactile stimuli to skin mechanoceptors and nerve free endings respectively); 6. Once learnt how to interpret the flow of afferent information, the user is able to infer size, shape and stiffness of the object held by combining, for example, the information relative to prosthetic hand aperture and force developed; 7. Such information can be used consciously or unconsciously to correct the new motor command (e.g., increase muscle contraction) without constantly looking at the device, thus freeing attentional resources

To provide the users with proprioceptive feedback from the prostheses, these should be equipped with appropriate sensors that extract information on positions and forces, as well as encoding algorithms and stimulators that convey the encoded pattern to the remaining functional sensory system of the user (Fig. 2). This complex process represents a great challenge, especially because of the low efficacy of the artificial interfacing system, flawed by: (i) the time consuming signal processing and transmission of information from the device to the user [42]; (ii) the area required to place all the components often disproportionally large compared to the available skin surface (*see below)* [43]; (iii) the repeated calibrations of the stimulation parameters, needed for a reliable user’s sensation and perception [44, 45].

Sensory restitution can be achieved through invasive or non-invasive strategies. We circumscribed our critical analysis to non-invasive strategies, excluding studies involving implantation surgery and manipulation of the patient’s anatomy [46, 47, 48].

In line with the literature, feedback is defined as *homomodal* when the artificial stimulus delivered to the user belongs to the same sensory system and modality conveying the missing information (e.g., conveying touch with devices that provide pressure feedback). On the contrary, *heteromodal* feedback or sensory substitution exploits a sensory channel that is different from the one employed physiologically (e.g., providing angular movement through hearing), or the same channel but changing the modality of the input stimulus (e.g., providing limb position through vibration in place of skin stretch).

Another important point relates to the body part used to restitute the feedback. Obviously, *homotopic* feedback—provided to the very same site of the body to which the information pertains- is not feasible in amputees due to the lack of the limb, thus *heterotopic* stimulation is mostly employed. An intriguing further possibility to approximate *homotopic* restitution is to exploit body territories that, when touched, provide sensations that the amputee refers to the lost limb [49, 50]. Such a referred sensation phenomenon, present in most amputees stump, neck and face, results from central and peripheral neural rearrangement [51].

Vibrotactile and electrotactile stimulation are among the most frequently used sensory substitution techniques [52, 53]. Vibrotactile stimulation is delivered by means of a mechanical vibration applied directly to the skin of the subject. The parameters of the vibratory stimulus (i.e. frequency and amplitude) can be independently modulated to convey different kinds of information [54, 55, 56, 57, 58, 59,60]. Single vibrators can be used independently or arranged in arrays, also giving the possibility to spatially encode the information to be delivered. Vibrators are usually low-power, unobtrusive and potentially embeddable within the prosthesis socket, worn upon the target stimulation area. Nevertheless, vibratory stimulation has several flaws, like habituation to the stimulus, which makes it barely perceivable after some time, and a low spatial resolution, because of its propagation to the surrounding tissues [61, 62].

Electrotactile stimulation involves an electric current delivered to the skin, inducing a local electric field that causes the afferent nerve endings to fire. It has many advantages, considering its stimulation parameters flexibility, the spatial resolution, and the small size of the available electrodes. However, the elicited sensation might turn into sharp and/or burning pain and thus need further calibration anytime the electrodes are replaced or even slightly displaced from their original location [63, 64, 65, 66, 67, 68, 69]. Without proper precautions (e.g., intermittent stimulation to be preferred to continuous stimulation), even this type of stimulation could suffer from the effects of habituation [70]. Because of the absence of moving mechanical parts, electrotactile devices require less power and respond faster than vibrotactile ones, ensuring shorter delays of sensory restitution. The integration of electro- and vibro-tactile stimulation, delivered simultaneously at the same skin location, was also tested [71], providing an effective example of how more sensory channels can be synchronously involved, reducing the overall skin area needed to convey more information**.**

Haptic devices capable to stretch or provide pressure to the user skin were also employed to deliver proprioceptive information [72, 73, 74], either in homomodal or in heteromodal feedback restitution. Skin stretch devices are typically accurate, with a good intensity range and resolution, but are also heavy, cumbersome and energy consuming, hampering their future integration in portable systems. Like vibrotactile devices, they suffer from a slow response of the system due to the mechanical inertia of the moving parts [53, 61].

Beside touch, other sensory modalities have been exploited for sensory substitution, such as hearing or vision [75]. Due to the strong association between auditory and motor areas [76], sonification can be used to improve motor and proprioceptive performances [77, 78, 79]. Afferent information can be encoded by modulating the pitch, timbre or volume of individual auditory signals or by employing combinations of different tones [80]. Auditory sensory substitution is light, fast, has low power-consumption, but it obviously interferes with normal hearing, is obtrusive and requires huge attentional resources. Another example of heteromodal sensory substitution is provided by augmented reality (AR) feedback, which allows to artificially increase the amount of visual information provided to the user by means of head-mounted displays or single-eye glasses as in [81]. Nevertheless, similarly to auditory sensory substitution, the interference with normal vision may become a limitation, not to mention the present need to wear additional devices on the face.

Proprioceptive homomodal restitution exploits muscle spindles and cutaneous mechanoreceptors to transmit information regarding the movements of a prosthesis to its user [61, 82]. Tendon vibration and skin stretch kinesthetic illusions can be elicited both at larger joints like the elbow [83], as well as at the smaller interphalangeal joints [25, 84]. However, while muscle spindles may remain after an amputation, such as the extrinsic muscles of the hand in transradial amputations, most of the times skin stretch must be delivered heterotopically [85], making the association between the feedback and the provided information less intuitive.

### Control loops involving proprioception

In the following lines, studies investigating proprioception restitution in prosthetics and closely related fields are analyzed, taking into consideration both the information extracted and encoded as well as the feedback strategy employed. The analysis addresses first the works involving only kinesthetic senses of position and velocity, as well as configuration of the grip, followed by studies in which also force feedback was investigated, mostly focusing on grip strength. The studies reported were selected through a literature search that included proprioceptive feedback and non-invasive stimulation methods. The involvement of proper prostheses in the experimental design was not a strict criterion for selection and also preliminary investigations involving virtual end-effectors or surrogates (e.g., cursors or lines on displays) were included given the pertinence to the topics and relative scarcity of works featuring a complete device. Although most participants involved in the studies were able-bodied subjects, some of the experiments described also included amputees. Table 1 summarizes the experimental set ups and the results of the reported studies.

**Table 1:** .

Authors (Year)	Participants	Efference: End effector and control	Encoded info	Sensory Feedback: Stimulation—localization	Evaluation methods and results
Mann & Reimers (1970) [55]	1 amputee	Myoelectric elbow prosthesis (Boston arm)	Elbow angle	Vibrotactile cutaneous display—Upper-arm socket	Improved accuracy (radial error) in matching tasks: comparable to standard mechanical prosthesis
Hasson & Manczurowsky (2015) [54]	27 able-bodied	Myoelectric virtual arm	Arm’s position and velocity	Vibrotactile (linear actuator)—Wrist of dominant arm	No improvements in end-point error, movement time and composite skill. In some cases, detrimental
Guémann et al. (2022) [87]	16 able-bodied and 7 amputees	Myoelectric virtual arm	Elbow angle	Vibrotactile array—1st third of upper arm/stump	Better performances in reach accuracy compared to no feedback. No further improvement with vision available but preferred according to NASA-TLX (workload evaluation questionnaire)
Marinelli et al. (2023) [88]	15 able-bodied and 1 congenital limb deficiency	Virtual wrist, manually controlled (keyboard keys)	Wrist angle (prono-supination)	Vibrotactile array (+ Gaussian interpolation spatial encoding)—Forearm	The novel method for continuous information encoding was demonstrated to be effective in providing the subjects with meaningful stimuli about prono-supination of the virtual wrist (average error < 10%). The approach described can be flexible customized in terms of sensation quality
Vargas et al. (2021) [89]	7 able-bodied	Myoelectrical finger	Finger MCP joint angle	Vibrotactile array—Upper arm	Subjects were able to control a myoelectric finger to the desired angle, both with a position- and velocity-based control, when provided with artificial proprioceptive feedback
Witteveen et al. (2012) [90]	15 able-bodied and 3 amputees	Virtual hand opening controlled by a scroll wheel	Hand opening (+ touch)	Vibrotactile vs Electrotactile—Forearm/Stump	In general, vibrotactile feedback performed better in grasping task considering duration, mean absolute error and % correct openings. Electrotactile feedback increases the duration. Addition of touch feedback further improves results
Garenfeld et al. (2020) [86]	13 able-bodied	Pattern classification-based Myoelectric control of virtual hand multiple DoFs	Wrist rotation and hand aperture	Electrotactile displays (spatial vs amplitude encoding)—Forearm (contralateral)	2 different feedback configurations delivered good performances in terms of completion rate, time to the target, distance error and path efficiency
Han et al. (2023) [91]	5 able-bodied and 2 amputees	No control [Passive discrimination]	Flexion–Extension of prosthetic wrist	Electrotactile (static and dynamic coding for position and movement senses)—Forearm/Stump	Subjects were able to discriminate among 5 different degrees of flexion–extension of the prosthetic wrist, as well as the transition between one position and another (temporal combination of multiple position sensations). Lower success rates were reported for movement recognition
Patel et al. (2016) [67]	11 able-bodied	Myoelectric control of virtual and multi-articulated hand	Hand configuration	Electrotactile—Forearm (contralateral to control muscles)	Feedback improved performances in matching task, compared to no feedback condition. Results when visual feedback is available are always better
Earley et al. (2021) [80]	16 able-bodied	Hybrid positional-myoelectric control of a 2 DoF prosthesis	Joint speed	Frequency-modulated audio feedback	Joint speed feedback reduced reaching errors in ballistic reaching task, only when the control was perturbed by doubling the EMG gain
Bark et al. (2008) [61]	10 able-bodied	Hand-held force sensor-controlled cursor on screen (virtual arm)	Cursor position	Vibrotactile/Skin stretch—Forearm	Both feedback strategies improved position error results. Overall, skin stretch performed better
Wheeler et al. (2010) [74]	15 able-bodied	Myoelectric virtual arm	Elbow angle	Skin stretch (rotational)—Upper arm	Lower targeting errors with feedback, compared to no feedback. However, proprioceptive feedback from the contralateral limb obtained the best results
Kayhan et al. (2018) [82]	11 able-bodied	No control [Passive discrimination]	Wrist multiple DoFs-related information	Skin stretch—Forearm	Feasibility of the approach demonstrated by means of confusion matrices for discrimination accuracy
Shehata et al. (2019) [99]	1 amputee (lower limb)	No control [Passive discrimination]	Ankle movement	Skin stretch + TVI—Stump	Combination of feedback strategies increased the consistency of the illusion by 30–40%, depending on the site where the stretch was applied, with respect to TVI alone. Skin stretch also increases range and speed of the illusory movement
Akhtar et al. (2014) [100]	5 able-bodied	Myoelectric virtual fingers	Finger joint angle/hand configuration	Skin stretch/Vibrotactile—Forearm	Subjects were able to discriminate 6 different grips with 88% accuracy. In 1 DoF virtual targeting task, performances were similar between skin stretch and vibrotactile feedback
Battaglia et al. (2017) [96]	18 able-bodied	Myoelectric prosthetic hand	Hand aperture (prosthesis DC motor encoder)	Skin stretch (linear)—Upper arm	The feedback device was successfully integrated with the myoelectric prosthesis. Subjects’ discrimination accuracy improved with respect to no feedback condition
Battaglia et al. (2019) [103]	44 able-bodied and 1 amputee	Myoelectric prosthetic hand	Hand aperture (prosthesis DC motor encoder)	Skin stretch (linear)—Upper arm	Feedback was effective even in conditions of higher cognitive load (distraction task). Improved performances were reported in a functional test (AM-ULA) and passive discrimination test with the amputee user
Rossi et al. (2019) [73]	43 able-bodied and 1 amputee	Myoelectric prosthetic hand	Hand aperture	Skin stretch—Forearm	Improved discrimination accuracy both in passive (hand resting on table) and active (actively moving the hand) settings. 75% accuracy was reported, against 33% in no feedback condition
Colella et al. (2019) [72]	10 able-bodied and 1 agenesia	Myoelectric prosthetic hand	Hand aperture	Skin stretch (“unidirectional” and “pinch”—Forearm/Stump	Similar discrimination accuracies with different configurations of skin stretch were reported
Pylatiuk et al. (2006) [106]	5 amputees	Myoelectric prosthetic hand	Grip force	Vibrotactile—Stump	Improved ability in regulating the grasping force, with a reduction of 37–54% reported, compared to a vision-only condition
Stepp & Matsuoka (2010) [107]	8 able-bodied	Phantom Premium 1.0 robotic device (Sensable Technology) for 3D monitoring of the index fingertip	Applied Force	Vibrotactile/Haptic—Upper arm	Addition of vibrotactile feedback resulted in increased virtual box displacement and decreased difficulty ratings compared to vision-only condition, but performances were poorer compared to the ones obtained with the direct haptic feedback provided by the robot. Increased task times were reported with vibrotactile feedback
Witteveen et al. (2014) [57]	10 able-bodied and 7 amputees	Virtual hand controlled by computer mouse scroll wheel	Hand aperture and grip force	Vibrotactile (position + amplitude modulation)—Different configurations: Forearm and entire upper limb/ Stump	Subjects provided with opening information alone or in combination with force feedback were able to discriminate 4 stiffness levels in 60% of the cases. Performances were significantly better than those obtained without feedback. Force feedback alone was not sufficient to discriminate stiffness. No statistical differences between hand opening info alone and in combination with force
Witteveen et al. (2015) [58]	10 amputees	Virtual hand controlled by computer mouse scroll wheel	Hand aperture and grip force	Vibrotactile (position + amplitude modulation)—Stump	Performances were similar to those reported in the able-bodied group in a previous study
Ninu et al. (2014) [105]	9 able-bodied and 4 amputees	Myoelectric prosthetic hand	Hand closing velocity and grip force	Vibrotactile—Forearm/Stump	Vibrotactile feedback was effective in replacing visual feedback. Force feedback was not essential for the control of grip, given that subjects were able to do so predictively by means of closing velocity
Jorgovanovic et al. (2014) [110]	10 able-bodied	Virtual 1 DoF hand prosthesis controlled through a single-axis contactless joystick	Grasping force	Electrotactile—Forearm	The results showed that subjects were able to learn and scale the force based on electrotactile feedback, resulting in a 72% success rate in grasping objects of different weights. The closed-loop control demonstrated robustness and improved performance compared to feedforward control
Chai et al. (2019) [104]	15 able-bodied	Single DoF myoelectric hand prosthesis	Grasping angle and force	Electrotactile—Upper arm (ipsilateral to control muscles)	The feedback allowed subjects to discriminate among 4 types of grasped object sizes (87.5%), 3 kinds of stiffness (94%) and 4 levels of grasping forces (73.8%)
Štrbac et al. (2016) [69]	10 able-bodied and 6 amputees	No control [Passive discrimination]	Grasping angle and force, wrist rotation and flexion	Electrotactile (spatial + frequency coding)—Forearm/Stump	Subjects were able to discriminate stimulation levels with more than 90% success rate. Amputee subjects demonstrated lower rates than able-bodied
Dosen et al. (2017) [64]	10 + 10 able bodied	Myoelectric + Virtual hand prosthesis	Grasping force	Electrotactile (spatial/spatial + frequency coding)—Forearm (contralateral to control muscles)	The study suggests that mixed (spatial + frequency) coding is a reliable method for transmitting high-resolution information and offers advantages in terms of compactness compared to other coding schemes. Despite psychometric differences however, the performance in closed-loop control tasks was similar for both coding schemes
Clemente et al. (2017) [81]	8 able-bodied	Data glove-controlled robotic hand	Grip aperture and force	Augmented reality	AR feedback was successfully integrated into subjects’ sensorimotor control loop. Participants were also able to decouple the two types of information provided. AR feedback allowed the subjects to execute the task more consistently, compared to no feedback condition
Dosen et al. (2015) [111]	10 able-bodied and 2 amputees	Myoelectric prosthetic hand	EMG + Force/Force	Visual interface	The addition of EMG information reduced twofold force dispersion. More accurate and stable tracking of force was also reported. According to authors, force was controlled predictively (given the anticipatory nature of EMG signal) and with a finer resolution
Schweisfurth et al. (2016) [68]	11 able-bodied and 1 amputee	Myoelectric prosthetic hand	sEMG envelope vs Force (prosthesis input vs output)	Electrotactile (spatial + frequency coding)—Forearm (contralateral to control muscles)	EMG allows for predictive control (improved feedforward control) and improved precision of myoelectric command and force control

#### Kinematic feedback: position, configuration and movement

**Fig. 3:**
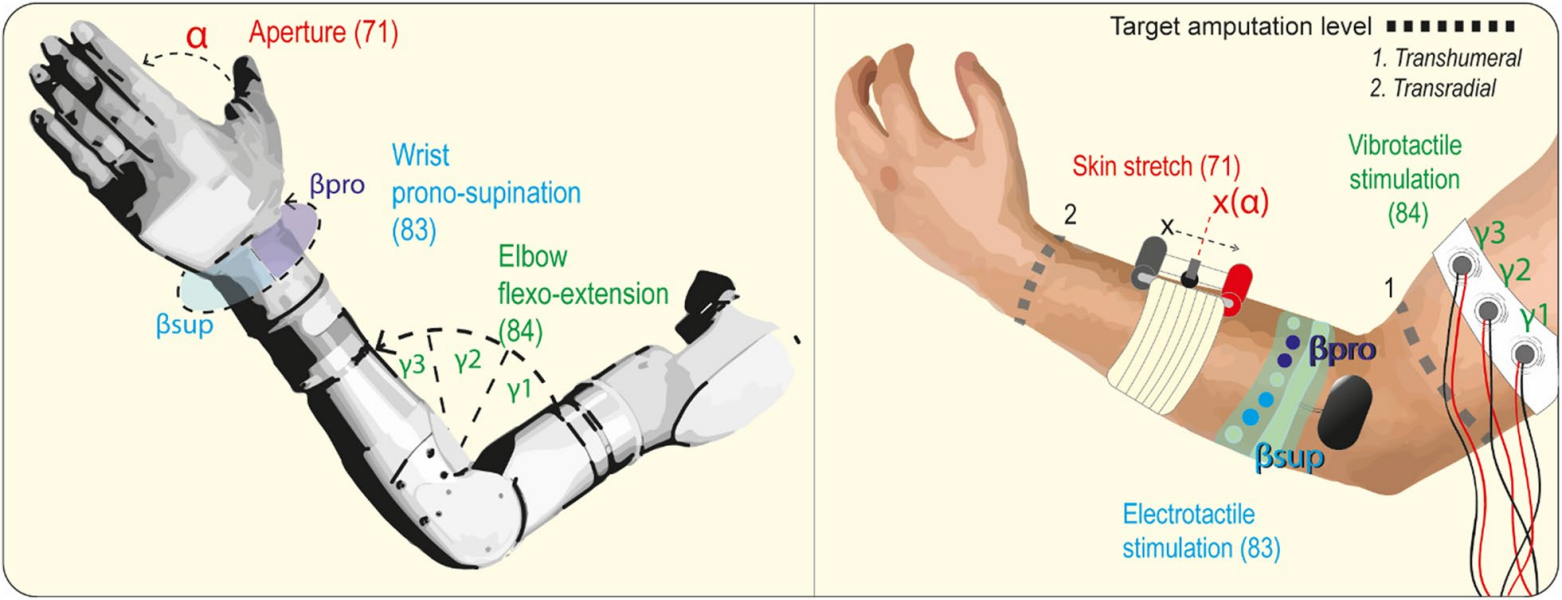
Examples of proprioception restitution strategies for coding positional and movement-related information. Stimulation devices have been illustrated as applied to a single limb to simulate their simultaneous use, but their choice will inevitably depend on the user’s level of amputation and will therefore be tailored to the individual. The degree of grip aperture has been encoded into the movement of a skin-stretch stimulation device, whose position on the user’s skin can be employed to infer the state of the prosthetic hand, thereby reducing the need for careful vision inspection [73]. Also, the prosthetic wrist’s prono-supination state has been fed back by activating a dedicated combination of electrodes on the user’s forearm [86]. Vibrotactile motors around the arm of the user have been used to encode discrete angular positions of the controlled myoelectric elbow [87]

Restitution of proprioceptive information regarding the position or the configuration of the prosthesis, as well as its movement, has been explored employing several feedback strategies (Fig. 3). One of the earliest attempts to restitute proprioception showed improvement of positional control of the myoelectric Boston Arm, granted by the addition of a vibrotactile array display providing elbow angle information [55]. However, contrasting results were reported later: in able-bodied subjects the addition of position- or velocity-based vibrotactile feedback did not increased the rate of skill acquisition (i.e., relationship between movement velocity and accuracy) or improved task performances. In some cases, it was, indeed, detrimental, given the better performances (decreased error and movement time) reported following the removal of the additional feedback [54]. More recently, similar performances between the vision-only and combined (vision + vibrotactile) feedbacks were reported, demonstrating that the vibration was not deteriorating the performances. However, the participants expressed, via the self-assessment workload evaluation NASA-TLX questionnaire, a 62.5% preference for the combined feedback, placing vision alone in second place [87]. Vibrotactile stimulation arrays, mounted around the forearm of the participants, have been also employed for providing the degree of wrist pronosupination. A novel and customizable approach was described by the authors, employing a variable number of vibration motors and a flexible Gaussian interpolation-based intensity encoding algorithm, that allowed the subjects to achieve < 10% average error in target-achievement tests [88]. Encouraging results were obtained when providing finger joint angle of a myoelectrical hand by means of a similar vibrotactile array mounted around the forearm of the user [89].

Electrotactile feedback also proved to be effective in returning proprioceptive information, even though vibrotactile stimulation was reported to provide better performances for hand opening feedback restitution in a virtual object grasping task [90]. The delivery of hand aperture and wrist rotation feedback using a compact 16-channels electrotactile interface was tested during myoelectric control in thirteen able-bodied subjects executing a target-reaching task. Participants were able to correctly perceive and interpret the two independent channels of electro-tactile stimulation delivered closely and simultaneously, allowing them to use a myoelectric interface to move a cursor to the required targets on two degrees of freedom (DoF) [86]. In a more recent instance, subjects were able to correctly identify the degree of flexion–extension of a robotic hand prosthesis as well as its movement from one position to another taking advantage of the feedback provided by a forearm electrotactile array using spatial coding for providing both static and dynamic types of information. High success rates were reported for able-bodied subjects and 2 amputees taking part to the experiments [91].

Individual fingers flexion level could also be encoded through electrotactile stimulation, allowing able-bodied subjects to reproduce a target level of flexion of either individual fingers or grips of a robotic hand actuated through myoelectric control. In this case, electrotactile feedback resulted to be better than no-feedback, but still, less useful than the visual-one [67].

Sonification has been employed in many motor control studies to improve proprioceptive performances, leveraging on intermodal learning and cross-modal processing [92], mostly dependent on the neuroanatomical interconnectivity between auditory and motor cortices [93]. When a properly tuned auditory feedback was associated to a movement, such movements were learnt faster and performed better [94, 95]. However, when sonification was specifically employed for proprioceptive sensory substitution, it yielded more modest results. Joint speed of a two DoFs virtual hybrid positional-myoelectric prosthesis has been encoded by modulating the frequency of an auditory feedback. Such feedback of the EMG-controlled DoF improved target-position reaching performances only when a perturbated control was introduced to the task [80].

Several devices exploiting the skin stretch channel have been developed to restore proprioception, providing a complex combination of velocity, timing, and static force sense which subjects can use to map position of the tracked end-effector [73, 74, 85, 96]. Positional feedback has been successfully provided in different settings, possibly implementing more DoF at the same time. A rotating skin-stretch device attached to the forearm was employed to encode positional information of a cursor’s movement and better performances were reported compared to vibrotactile feedback [61]. According to the authors, skin-stretch provided a more intuitive mapping for position information and a more realistic sense of velocity. In their following study, the same device was similarly used to convey feedback of a virtual object movement, in both active positioning and passive perception tasks. The subjects were able to map the feedback to the movement with minimal training, but poorer results were reported in the passive perception task, where the need of a higher level of concentration was reported [85]. Angular position of a virtual EMG-driven prosthetic elbow was encoded into the magnitude of the provided skin stretch, leading to an overall improvement of the performance in a blind targeting task when compared to no-feedback control condition [74]. Skin stretch can also be used to deliver more complex information using more than one actuator, e.g., rotation and translation of a robotic limb [97, 98]. A skin stretch tactor was designed to provide homomodal feedback about the three DoF wrist movements, based on the virtual angular position. Subjects were able to correctly identify the corresponding positions of the end-effector, even if prono-supination and ulnar-radial patterns of skin deformation, possibly similar, were sometime confounded [82]. Skin stretch has been also successfully integrated with TVI to provide information about the ankle angle to a lower limb amputee [99].

Skin-stretch feedback has been employed to convey information on hand aperture. No significant difference was shown between skin-stretch and vibrotactile feedback when participants were asked to classify six different hand configurations after a short training [100]. Despite the similar performances, in the perspective of a prosthetic applications, the authors reported that their skin stretch device was more efficient in terms of power consumption, surface area occupied and weight. The Rice Haptic Rocker [96], an evolution of previously developed skin stretch devices [97, 98, 101], employs a rubber pad stretching the upper limb skin proportionally to the gripper aperture. The skin stretch feedback improved the performances in an object size discrimination test and provided feedback intuitive enough to not require significant dedicated attention. The device was also evaluated on a trans-radial experienced myoelectric prosthesis user who performed better on the Activities Measure for Upper Limb Amputees (AM-ULA) [102], a measure evaluating task completion, speed, movement quality and skillfulness of prosthetic use. More modest results were reported in the passive size discrimination test [103]. Promising results in discrimination tasks, both in able-bodied subjects and one amputee, were reported employing the Hap-pro, a similar device featuring a moving wheel on the user’s forearm [73]. A further alternative is represented by the Stretch-Pro, which through the inward and outward rotation of one or two actuators allows a physiological-like deformation of the skin to be associated to movements. It outperformed the Hap-pro (85% vs 77% average accuracy) in the same discrimination task [72].

#### Kinetic feedback: addition of force

In a closed-loop control scheme, regulation of grasping force could benefit from proprioceptive-like feedback because visual clues are often not informative enough, even when constant visual monitoring is dedicated to the artificial hand.

There is still contrasting evidence about the benefits of providing hand force-related proprioceptive information through vibrotactile feedback. A study reported improved performances, where grip force feedback helped in reducing the effort of experimental subjects that were otherwise applying excessive and unnecessary force in the vision-only condition [106]. Vibrotactile feedback of force added to a virtual object manipulation task improved performances and decreased difficulty ratings with respect to control condition featuring only visual feedback [107]. In further studies, force feedback was reported to be unhelpful, except for a few individuals more experienced with myoelectric prostheses [108, 109].

**Fig. 4:**
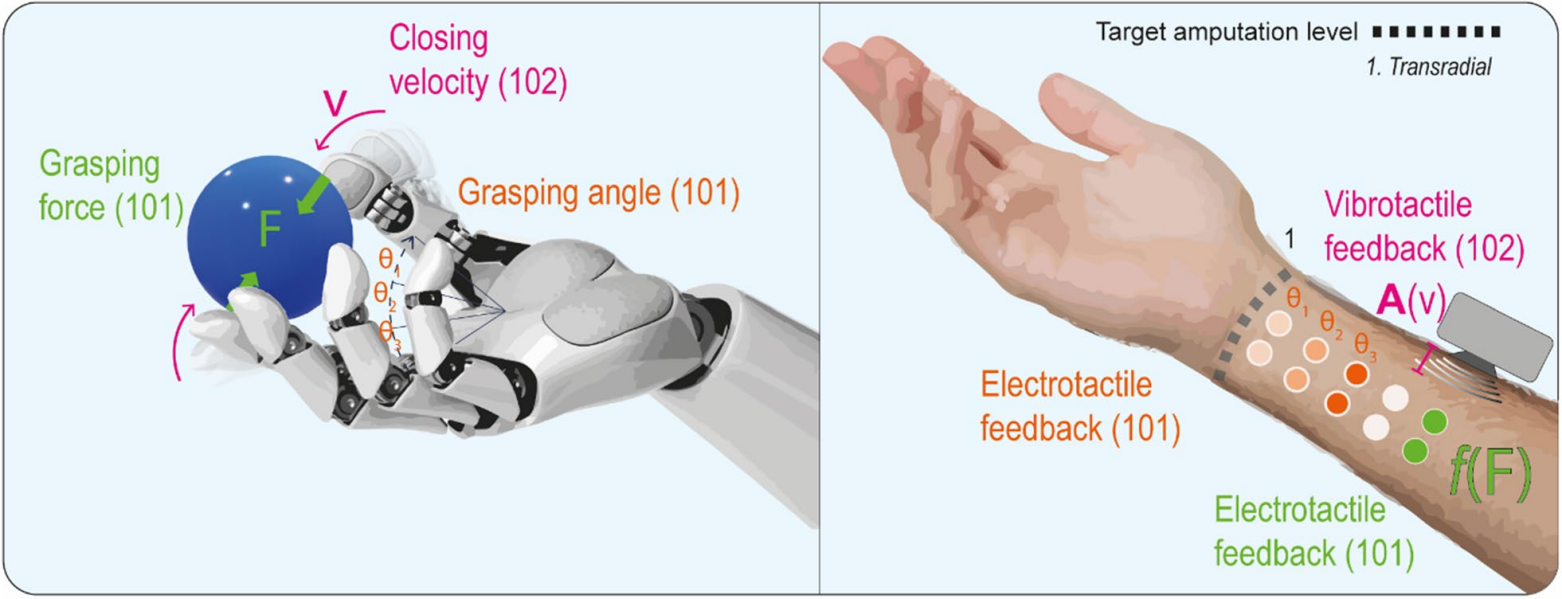
Examples of proprioception restitution strategies integrating both kinematic senses of position and movement and sensation of force. Stimulation devices have been illustrated all together, although the choices of the devices as well as eventual combinations must be tailored to the individual’s needs. Electrotactile arrays can be used to provide different types of information by means of multiple encoding strategies: for instance, discrete grasping angles, corresponding to specific couples of electrodes on the user’s forearm can be conveyed through spatial coding. Additionally, the frequency of the electrotactile stimulus provided by the last pair of electrodes can be proportionally adjusted to reflect the grasping force measured at the tips of the prosthetic fingers [104]. Using a different approach, the amplitude of a vibrotactile stimulus on the user’s forearm was set proportional to the grip’s closing velocity [105]

A vibrotactile array on the forearm was employed to relay the single DoF of a virtual hand grip aperture and grasping force in able-bodied subjects and, later, in amputees [57, 58]. The use of this feedback allowed an improvement of grasping performances compared to no-feedback condition, but not compared to the condition when visual feedback was also available. A further study evaluated object manipulation performances after providing closing-velocity and grasping force, visually or through vibrotactile stimulation [105] (Fig. 4). Direct force feedback did not prove to be essential, since grip strength could be controlled predictively, estimating it from the closing velocity.

Electrotactile stimulation has also been used to return information about the force applied by a controlled device. Grasping performances of ten able-bodied subjects were tested in a virtual environment: two bipolar concentric electrodes, individually tuned according to perception and pain thresholds, delivered the same (either grasping or lifting) force information to participants who had to perform a virtual grasp and lift task using a joystick as a gated ramp controller (the type of control and feedback changed from grasping to lifting by pushing a button). Participants performed well also in sessions with novel objects, demonstrating a successful skill learning and transfer [110]. In another study, the force and grasping angle of a single-DoF myoelectric hand prosthesis were simultaneously encoded through spatial and intensity modulation of a five-channels electrode array stimulator, allowing able-bodied participants to discriminate 4 object sizes, 3 degrees of softness and 4 levels of grasping force [104]. More complex information encoding has also been attempted. A compact multichannel electrotactile interface and a set of pre-programmed stimulation patterns were tested with the aim to translate aperture, grasping force and wrist rotations of a multi-DoF prosthesis [69]. Tests were performed in ten able-bodied participants, and in 6 amputees to successfully prove the feasibility of this approach. The same research group tested the system in force control with routine grasping and force tracking tasks, using both a real and a simulated prosthesis in healthy subjects. Simultaneous spatial and frequency encoding obtained the best results in preliminary psychometric tests, proving to be the best method to reliably transmit up to 15 levels of high-resolution proprioceptive information. Results in the routine grasping task were similar between the benchmark visual feedback and the electrotactile feedback [64].

Using augmented reality (AR) feedback, both proprioceptive information of grip closure and force of a robotic hand were provided by proportionally scaling the horizontal and vertical axes of an ellipse shown in the display [81]. Although such information was redundant and not strictly necessary to complete the grasping task, the addition of the AR feedback resulted in more consistent performances across the trials. Moreover, such feedback induced subjects to modify their behavior (they scaled the grip force according to the modified corresponding axis of the ellipse), demonstrating the integration of the feedback into the sensorimotor dynamics.

Finally, an interesting alternative to force feedback is to provide the user with the information about the myoelectric system input, that is, the EMG activity itself. Performances were, indeed, improved when EMG feedback was added to force feedback both by decreasing force dispersion during routine grasping and increasing accuracy and stability in tracking the reference force profiles during a force steering task [111]. A later work by the same group, compared the two types of information separately, conveyed through an electrotactile interface, and reported both an improved precision and decreased absolute error in the generation of grasping forces for the EMG feedback [68]. EMG-feedback is anticipatory in nature, given that the input signal is generated much before the final output of the system (e.g., speed or force) and it gives the user time to adjust its behavior, explaining the obtained results.

## Discussion and conclusion

The analyzed studies do not highlight a best approach to provide non-invasive proprioceptive feedback. All the stimulation techniques described exhibit both pros and cons. On one side, homomodal feedback restitution (e.g., skin stretch) offers a more intuitive way of relaying information to the user, but there could be still some obstacles to the integration of these types of stimulators within prosthesis sockets (e.g., bulky size, high power consumption). On the other, heteromodal stimulation is less intuitive than homomodal techniques, but its broad applicability and cost-effectiveness, as well as the greater freedom to change stimulation features (e.g., independent modulation of amplitude and frequency of vibrotactile and electrotactile stimulation, the possibility to arrange multiple stimulators in arrays), make it a valuable option for feedback restitution. Also, it may be argued that heteromodal proprioception restitution takes advantage of predominantly exteroceptive sensory channels (e.g., skin mechanoceptors and nerve free endings). However, proprioception and exteroception are intertwined, and the boundary between the two is blurred, with information from multiple receptors being integrated at multiple levels. We can perceive the external world through our proprioceptors (e.g., translating the configuration of the hand into the size of a grasped object), and skin mechanoceptors have a dual nature, providing information about both movement and contact forces. Therefore, as long as the sensory stimulus can be translated into meaningful information regardless of the input channel, heteromodal stimulation should be considered as valuable as the homomodal counterpart [112].

Regarding the results obtained with each stimulation technique, there is evidence to support the use of vibrotactile feedback for the restitution of proprioception. More accurate performances, compared to other feedback conditions, were reported when relaying both hand configuration and grip force [106, 109]. Skin stretch stimulation, also, appears to be a reliable way of proprioceptive information transfer, providing more consistent and unambiguous results [73, 85, 103]. Beside the more intuitive nature, the association between movement and a skin stretch stimulation may be more direct since both can be generally described with a modulus (stimulus intensity) and a direction.

Electrotactile feedback has proven to be another viable alternative for proprioceptive feedback restitution. Many channels can be placed on a limited surface, as the one of the amputee stump, and more advanced coding schemes can be employed [64]. This likely allows to deliver more types of information simultaneously, a convenient feature for the restitution of proprioception that is composed of multiple types of information merged together. Still, care should be taken to electrodes repositioning and all the issues related to changes in perception and pain thresholds [44]. Both AR and auditory proprioceptive feedbacks need further development and refinement to go beyond research settings. They interfere with normal sight and hearing and may require the user to devote non-negligible attentional resources, unlike normal proprioception which contributes to motor control in a mostly unconscious way. While it is true that other types of stimulation would similarly require the user’s attention, peripheral input channels (e.g., the skin of the stump) are not at risk of being saturated with information as central senses such as sight and hearing are.

Furthermore, it should be noted that different types of stimulation strategies may vary in their suitability for restoring proprioception in multi-articulated prostheses. Each strategy exhibits varying levels of efficiency in handling different numbers of DoF. Electrotactile arrays have the advantage of compactly encoding a larger amount of information related to multiple degrees of freedom within a limited space compared to other devices like skin stretch devices. The latter, despite the higher energy requirements, could indeed perform better in single-DoF scenarios, given, for example, the greater familiarity of subjects with the stimulation, but it may no longer be optimal for multiple-joint/DoF control.

Skin receptors serve as the primary pathway for information transmission in most sensory substitution approaches. In the case of amputees, the availability of tissues may vary, but it remains evident that targeting regions with the highest density of receptors, thus providing the highest resolution for perception, would be the most straightforward strategy. This can be considered true regardless of the specific type of information being encoded. In humans, beside the face whose stimulation would be invasive and impractical, such high density is found distally along the limbs, with the highest found in the skin of the hands and fingers, which, in most amputation cases requiring the use of a prosthesis, is absent. Therefore, in the literature, the remaining tissues of the upper limbs (e.g., forearm and arm), represent the preferred choice for sensory substitution studies [52]. Moreover, it can be speculated that the proximity between the source of sensory information and the muscles controlling the end effector to which the information pertains facilitates the sensorimotor integration required for optimal control of the latter. This is due to the somatotopic cortical organization which plays a role in enabling sensorimotor integration, that, indeed, relies on intrareal neuronal coherence [113], which has been shown to depend on the spatial reach [114]. In case of homomodal stimulation (e.g., TVI), the localization strategy must be dictated by the type of perception you want to elicit. It is worth also considering that, beside the location, also the preferred pattern of stimulation may vary among different subjects. For amputees, this will be also affected by the remaining tissues of the stump, both in terms of quantity and quality of innervation. It is therefore fundamental to be able to adjust such patterns on a patient-specific basis [115].

Further technological development of the hardware (e.g., reduced dimensions, lower power consumption, better performing encoding algorithms) is likely necessary to achieve a completely satisfactory feedback strategy. However, more tools to investigate all the effects deriving from the additional feedback are needed to help define such a degree of satisfaction. Thus, to this aim, in the following paragraphs some considerations are made on the possible aspects of proprioception restitution to be investigated.

When evaluating the effects of an additional feedback, two strategies can be adopted, one after the other or in parallel: (i) single-modality evaluation; in the preliminary phases of developing a restitution strategy for a specific sensory channel, it may be beneficial to separately evaluate the contribution of individual feedback modalities (e.g., vision, hearing, natural/artificial proprioception etc.); (ii) integrated evaluation; considering that the aim of feedback restitution is the improvement of the prosthesis’ control in real life, the experimental settings should also allow to simulate and evaluate the effects of the ecological integration of multisensory information. It is important to consider that some advantages of the added feedback may be intangible in one case, but extremely significant in the other and vice versa.

In most of the reviewed works, however, the artificial feedback (e.g. vibrotactile) was only compared to a no-feedback condition [55, 74] and in the few cases where the addition of proprioception to vision was tested, performance did not improve [116]. There are at least two potential reasons for this: (i) ceiling effect, i.e., the experimental task is so easy that visual supervision is enough to execute it at its best, and thus, more ecological tasks are needed, designed so that their effective completion requires rich and multimodal adaptive behaviors; (ii) need for longer training. Indeed, the advantages of the added feedback might not be evident after a short learning phase. Humans are accustomed to experience the environment and its unpredictability through vision, relying predominantly on it [117, 118, 119, 120] so that integrating the new artificial feedback into the sensorimotor dynamics, as well as learning how to properly use it, may likely require a long time. Once accustomed to the new feedback, the relative weight of the proprioceptive information in building the motor command should increase and influence the performances [117]. In a few studies where the users where given enough time to train and learn, performances over multiple sessions were indeed improved by the proprioceptive feedback [121, 122]. Furthermore, as the subjects’ ability to determine relevance and effectively utilize new information improves, their perception of the stimulus itself has also been demonstrated to enhance through training. For instance, training has led to improvements in the detection of subtle intensity differences, resulting in a more effective transfer of information, given the increased resolution of perception [123].

Nevertheless, the potential enhancement of real prosthetic device control through the incorporation of proprioceptive sensory feedback systems may vary. Existing literature suggests a significant improvement compared to complete feedback deprivation. The regularity in a prosthetic device’s operation across various activities is an additional key factor to consider when gauging its control quality. Indeed, restoring proprioception has been shown to reduce the variability in the device’s performance [124]. Moreover, while vision plays a dominant role in simple tasks involving a single degree of freedom, the coordination of multi-joint movements is more likely to rely on proprioceptive inputs for efficient control [125] and, therefore, proprioceptive feedbacks for the actuation of more complex prosthetic devices.

Proprioceptive feedback, beside improving motor control, has also the potential to enhance the embodiment of the prosthesis, a complex, multi-componential process referring to the sense of owning and controlling our own body parts, whether real or artificial [126, 127]. Embodiment builds up from the inter-sensory congruency of stimuli [128], which could be further enhanced by the similarly congruent addition of proprioceptive feedback. The artificial feedback can be congruent with the visual feedback coming from the moving end-effector to be embodied [129, 130], as well as with the efferent motor command (i.e., efference copy) that can be treated itself as an additional sensory modality [84]. Considering that the myoelectric control of a device contributes to its embodiment [131], supporting the role of visuomotor congruency in its induction [132], we hypothesize that further enriching the visual feedback with proprioception would provide a collateral ownership-boosting congruence, likely beneficial to the device acceptance. Hitherto, in prosthetics, the effects of proprioceptive feedback on embodiment has not been investigated as extensively as, for instance, the addition of touch [49, 71, 133, 134, 135]. To the knowledge of the authors, a limited number of works studied the embodiment together with motor performances, but promising results were obtained using peripheral nerve stimulation providing both touch and kinesthetic sensations [136], and by using the TVI in amputees who underwent nerve-transfer surgery [137] (Targeted Muscle Reinnervation, i.e., surgery involving the transfer of motor and sensory nerves from the stump to remaining functional body parts in order to use the latter to amplify motor signals and feed them into myoelectric interfaces [138]).

Acceptance of the prosthesis depends also on its ease of use, considering both physical and mental effort. So far, addition of sensory feedback was mostly discussed in terms of performance improvement but, when assessing its usefulness, motor performance may not reflect the complete experience of the amputee. Controlling a prosthesis that does not provide feedback on its configuration and motion requires the user to devote continuous visual contact to its operation, with a consequent burden of attentional resources. One of the purposes of additional feedback should be precisely, when performing any task, to reduce the required cognitive load (i.e., a measure of the total amount of mental effort that an individual must exert in order to complete a task, including both the effort required to process new information and the effort required to maintain that information in working memory [139]). For instance, in a simple cup transfer task the addition of kinesthetic feedback allowed the subjects to “trust” the prosthesis more, thus influencing the time-percent fixation to the hand and to the target, resulting in a control behavior and attentional commitment closer to the one of able-bodied subjects [137]. Vibrotactile feedback relaying the force exerted by a myoelectric prosthesis, compared to a no-feedback condition, led to a reduction of the hemoglobin concentration in the right medial prefrontal cortex measured with functional near-infrared spectroscopy (fNIRS), suggesting a reduced mental effort needed to operate the device [140]. Further physiological parameters, like encephalic and cardiac electrical activity (EEG, ECG), electro-dermal activity (EDA), and respiration rate, were also shown to be good predictors of the cognitive load associated to manipulation tasks [75], and could serve our purpose.

Methods to assess brain plasticity resulting from proprioception manipulation could further support behavioral results with more objective and quantifiable parameters. Proprioceptive feedback, indeed, especially when it is experienced along a training period, is able to modulate brain motor [141, 142] and somatosensory [143] functioning. Components of somatosensory evoked potentials (SEPs) have been suggested as indices of proprioceptive afferences [144, 145, 146, 147] and could be used as evidence of the artificial input’s influences on cognitive processes. Furthermore, internal models are continuously updated by proprioceptive inputs [148] and provide the starting point for motor planning. Motor evoked potentials (MEPs) are a suitable indicator of the dynamic changes occurring during the preparation of an action [149] and could therefore represent a tool to objectify the effects of artificial proprioception on motor behavior.

Lastly, due to the easiness of recruitment and the need to avoid the premature involvement of patients to test preliminary hypotheses, most of the analyzed studies on sensory substitution have been conducted on able-bodied subjects. Studying healthy subjects is extremely useful, but the generalization of the results to amputees should be approached with extreme care, especially because of the plastic phenomena affecting amputees’ brain (extensively covered elsewhere e.g., [51, 150]). Plastic changes of their sensorimotor circuits, believed to be at the basis of the phantom limb pain (PLP), are particularly relevant for the study of sensory substitution [151, 152]. PLP is the sensation that the lost part is still present and painful, experienced by most amputees [153]. Several cues suggest that proprioception has a key role in PLP [154, 155, 156]. While some amputees can move their phantom at will, in the majority of cases the phantom is locked or frozen in fixed positions usually resembling the position of the limb just prior to the amputation. This has been interpreted as the result of some kind of persisting “proprioceptive memory” [154]. The contribution of proprioception in PLP is also suggested by Ramachandran and Hirstein (1998), whose foundational work proposed a mismatch between visual and proprioceptive inputs as a major contributor to the syndrome and provided the rationale for “mirror therapy” [157]. Indeed, mirror therapy aims at artificially restoring the congruency between visual feedback (the reflection of the healthy limb), the copy of the produced motor command (the efference copy) and the proprioceptive re-afferents (due to stump muscles' activation). With the same purpose, vision can also be tricked with an healthy-looking limb through videos and Virtual/AR [158]. The improvement of PLP and the reduction of abnormal cortical plasticity after a long-term use of myoelectric prosthesis [159] is likely based upon the same rationale. The lack of the congruent proprioceptive information from the artificial limb that the user controls and sees could represent a defective opening within the ideal closed loop of prosthetic motor control that deserves to be further investigated in the future. In light of the preceding discussion, beside the integration of the device in the body schema of the user (see paragraph on Embodiment), care should be taken, whenever the recruitment makes it possible, to the effects of artificial proprioception on cortical plasticity and phantom limb phenomena [155], since both have been shown to depend heavily on multisensory congruence.

## Data Availability

Not applicable.
